# Analysis of Th1/Th2 cytokine profile and clinical characteristics of patients with head and neck squamous cell carcinoma

**DOI:** 10.17305/bb.2024.10783

**Published:** 2024-12-01

**Authors:** Rong Wang, Gaofei Yin, Wei Guo, Nuan Li, Yang Zhang, Xiaohong Chen, Zhigang Huang

**Affiliations:** 1 Department of Otolaryngology-Head and Neck Surgery, Beijing Tongren Hospital, Capital Medical University, Beijing, China

**Keywords:** Cytokine (CK), Th1/Th2 cytokine, head and neck squamous cell carcinoma (HNSCC), interleukin (IL)-1β, interferon (IFN)

## Abstract

Immune system biomarkers in cancer pathogenesis offer new therapeutic avenues, as seen in cytokine (CK) profiles of laryngeal and hypopharyngeal tumors. A retrospective analysis was conducted on 58 patients with laryngeal and hypopharyngeal tumors and 27 patients with benign vocal cord lesions to explore the role of serum CKs in these diseases’ characteristics and immunotherapy. The differences in the levels of 12 CKs were measured. Additionally, the study examined the correlation between T helper cells (Th)1/Th2 CK levels and the clinical characteristics and immunotherapy efficacy of laryngeal and hypopharyngeal cancers. The results show that the balance of Th1/Th2 is biased toward Th2 in patients with laryngeal and hypopharyngeal tumors. Among these, Interleukin (IL)-6 (*P* ═ 0.021) was highly expressed in laryngeal tumors, and the expression levels of IL-1β (*P* ═ 0.008), IL-6 (*P* ═ 0.005), and IL-8 (*P* ═ 0.05) were higher in patients with poorly differentiated laryngeal tumors. The level of IL-4 (*P* ═ 0.0048) was significantly correlated with tumor location. The expression levels of IL-2 (*P* ═ 0.010), IL-4 (*P* ═ 0.028), IL-10 (*P* ═ 0.011), IL-12p70 (*P* ═ 0.034), IL-17 (*P* ═ 0.024), tumor necrosis factor (TNF)-α (*P* ═ 0.003), and interferon (IFN)-γ (*P* ═ 0.007) were related to lymph node metastasis. The level of IFN-γ (*P* ═ 0.016) was correlated with the efficacy of neoadjuvant therapy, while the level of IFN-α (*P* ═ 0.013) was significantly correlated with programmed death ligand 1 (PD-L1) expression. The Principal Component Analysis (PCA) results showed that patients with tumors, poor differentiation, and lymph node metastasis had higher levels of Th1 and Th2 CK separation. In conclusion, the shift in the balance of Th1 and Th2 CK expression indicates higher tumor invasiveness, and IFN has potential as a circulating molecular marker for immunotherapy of head and neck squamous cell carcinoma.

## Introduction

Head and neck squamous cell carcinoma (HNSCC) is a widespread cancer, ranking as the sixth most common globally. Due to its subtle and highly invasive nature, approximately 60% of patients are diagnosed in the later stages of the disease, resulting in a generally poor prognosis [1]. Extensive research in various areas has advanced our understanding of tumor immunity, leading to the development of immunotherapy. However, only a small percentage of patients with advanced pharyngeal squamous cell carcinoma benefits from this treatment, with approximately 20%–30% seeing actual improvements from immune checkpoint inhibitors (ICIs) alone. Therefore, further comprehensive and accurate research is necessary to better understand the tumor immune status of HNSCC patients.

Cytokines (CK) can have both stimulating and inhibitory effects on malignant tumors, depending on which receptors they bind to and which signaling pathways they activate. Additionally, they have a regulatory function within tumor immune microenvironments, making them possible biomarkers for predicting disease susceptibility. This study examines the relationship between serum CK levels and clinical characteristics in patients with laryngopharynx squamous cell carcinoma and investigates the impact of CKs in HNSCC.

## Materials and methods

### Patient information

Data was collected from patients admitted to the Department of Otolaryngology, Head and Neck Surgery at Beijing Tongren Hospital (affiliated with Capital Medical University) between January 1, 2023, and August 1, 2023. This included 58 patients with laryngopharyngeal squamous cell carcinoma and 27 patients with benign vocal cord lesions (vocal cord leukoplakia/polyps). Serological CK tests were performed on 85 patients to measure serum levels of 12 CKs, including interleukin (IL)-1β, interleukin-2 (IL-2), interleukin-4 (IL-4), IL-5, interleukin-6 (IL-6), IL-8, interleukin-10 (IL-10), IL-17, IL-12p70, tumor necrosis factor (TNF)-α, interferon (IFN)-α, and IFN-γ. These CKs were categorized into Th1 CKs: IL-2, IL-12p70, IFN-γ, IFN-α, TNF-α; And Th2 CKs: IL-4, IL-6, IL-5, IL-8, and IL-10. Demographic information was also collected, including age, smoking history, drinking history, tumor location, differentiation degree, AJCC stage, lymph tissue metastasis, and treatment methods.

### Cytokine (CK) detection

The 12-in-1 CK detection kit from Qingdao Resker Biotechnology Co., Ltd., was used to evaluate serum samples from patients using a multiplex microsphere flow immunofluorescence assay. All operations are strictly carried out in accordance with the product manual. Samples were prepared by combining 25 µL of serum with 75 µL of sample solution (comprising 25 µL each of measuring buffer, capture microsphere antibodies, and test antibodies for IL-1β, IL-6, interleukin-12 (IL-12), IL-17, IL-8, IL-5, IL-2, IL-4, IL-10, TNF-α, IFN-α, and IFN-γ). This mixture was incubated at room temperature (25 ± 1 ^∘^C) in the dark, without vibration, for 2 h (approximately 400–500 rpm). After adding 25 µL SA-PE, incubation continued under the same conditions for 30 min. Tubes were then washed with 1000 µL buffer, centrifuged at 300–500 ×*g* for 5 min, and the supernatant was discarded. Tubes were inverted to dry on the absorbent paper. The microspheres were resuspended in 150–300 µL washed by buffer and vortexed for 10 s, and samples were analyzed immediately using flow cytometry.

### Neoadjuvant therapy

The patient received neoadjuvant therapy consisting of a PD-1 inhibitor combined with chemotherapy for a total of 1–2 cycles. At the start of each cycle, an intravenous injection of 200 mg of PD-1 inhibitor pembrolizumab was given on the first day. The chemotherapy regimen included paclitaxel and nedaplatin, with 210 mg of paclitaxel being administered intravenously on day 2, and 40 mg of nedaplatin being given from day 3–5. The effectiveness of the immunotherapy in solid tumors was evaluated using internationally recognized criteria (RECIST 1.1) through imaging examinations. Complete response (CR) was determined by the disappearance of all target lesions or a reduction in size to less than 10 mm lasting for at least four weeks. Partial response (PR) was defined as a decrease of at least 30% in the sum of the diameters of all measurable target lesions compared to baseline, lasting for four weeks or more. Progressive disease (PD) was identified as an increase of 20% or more in the minimum value of the sum of all target lesion diameters, with an absolute value increase of 5 mm or more or the appearance of new lesions, despite not achieving complete or partial remission. Stable disease (SD) indicated disease stability, with changes in lesion volume and quantity between partial remission and disease progression.

### Immunohistochemistry

For PD-L1 antibody detection and combined positive score (CPS), tissues were fixed in 10% neutral buffered formalin at room temperature (15 ^∘^C–25 ^∘^C) for 6–72 h. The VENTANA PD-L1 (SP263) Assay was employed using the BenchMark ULTRA system. Tissues were processed by dewaxing at 60 ^∘^C for 12 min, followed by antigen retrieval with Cell Conditioning Solution (CC1) for 64 min. VENTANA PD-L1 (SP263) was used as the primary antibody, incubated at 36 ^∘^C for 16 min. This was followed by an 8-min incubation with OptiView HQ Linker and Multimer. Staining was completed with Hematoxylin II for 4–8 min and a blue reagent for 4 min to achieve the final immunohistochemical visualization.

For P53, P16, P63, and Ki-67 detection using the PV-6000 kit, paraffin-embedded sections were dewaxed and rehydrated. Endogenous peroxidase activity was quenched by incubation in 3% H_2_O_2_ for 5–10 min at room temperature. Sections were washed and soaked in PBS for 5 min, followed by antigen retrieval in a pressure cooker for 2.5–3 min. Primary antibodies were applied and incubated at 37 ^∘^C for 1–2 h or overnight at 4 ^∘^C. After washing, a secondary antibody from the PV-6000 kit was added and incubated for 30 min at room temperature. Visualization was achieved with DAB and sections were then washed, counterstained, dehydrated, cleared, and mounted.

### UALCAN analysis

UALCAN is a comprehensive and interactive web resource that provides easy access to publicly available cancer omics data (http://ualcan.path.uab.edu/index.html). Here, the mRNA and expression for genes *IL-1β*, *IL-6*, *IL-12*, *IL-17*, *IL-8*, *IL-5*, *IL-2*, *IL-4*, *IL-10*, *TNF-α*, *IFN-α*, and *IFN-γ* in HNSCC was evaluated using TCGA databases.

### Ethical statement

All methods were conducted in accordance with relevant guidelines and regulations. Prior to participating in the study, all patients or their legal guardians provided written informed consent, and for minors, parents or legally authorized representatives provided written informed consent. The Ethics Committee of Beijing Tongren Hospital (TRECKY2021-050; TREC2023-KY009) approved the study.

### Statistical analysis

Statistical analysis was performed using SPSS version 24.0 and R version 4.0.2 (available at https://www.R-project.org). Variables with a normal distribution were analyzed using independent sample *t*-tests, while categorical variables were examined using the chi-square test. Correlations and effects between various CK levels and clinical features were estimated. All *P* values were two-tailed, with *P* < 0.05 considered statistically significant. Principal component analysis (PCA) was conducted using the “limma” and “scatterplot3d” packages in R for analysis and visualization.

**Table 1:** General information

**Clinical feature**	**Grouping**	**Cancer (58)**	**Benign (27)**
Age	≤45	2	12
	(45–60]	22	10
	(60–75]	28	0
	>75	6	5
Sex	Female	1	3
	Male	57	24
Smoking	Yes	49	11
	No	9	16
Alcohol consumption	Yes	36	8
	No	17	19
Tumor site	Laryngeal	33	27
	Hypopharyngeal	25	0
Differentiation	High	29	–
	Low	29	–
Stage	I	13	–
	II	10	–
	III	13	–
	IV	21	–
Lymph node metastasis	Yes	25	–
	No	32	–
P53	+	35	–
	−	23	–
P16	+	5	–
	−	53	–
P63	+	33	–
	−	25	–
Neoadjuvant therapy	Yes	11	–
	No	47	–

## Results

### General information

The study included a total of 85 patients, consisting of 58 patients with pharyngeal cancer and 27 patients with benign vocal cord lesions (Table 1). Of these, 33 patients had laryngopharyngeal squamous cell carcinoma, with 33 cases of laryngeal cancer and 25 cases of hypopharyngeal cancer. The median age of this group was 63.5 years. Among them, 11 patients received preoperative anti-PD-L1 therapy combined with chemotherapy, while 47 patients underwent direct surgical treatment. The remaining 27 patients had benign vocal cord lesions, such as vocal cord keratosis, polyps, or dysplasia.

### Correlation analysis between serum CK levels and different diseases

**Figure 1.**
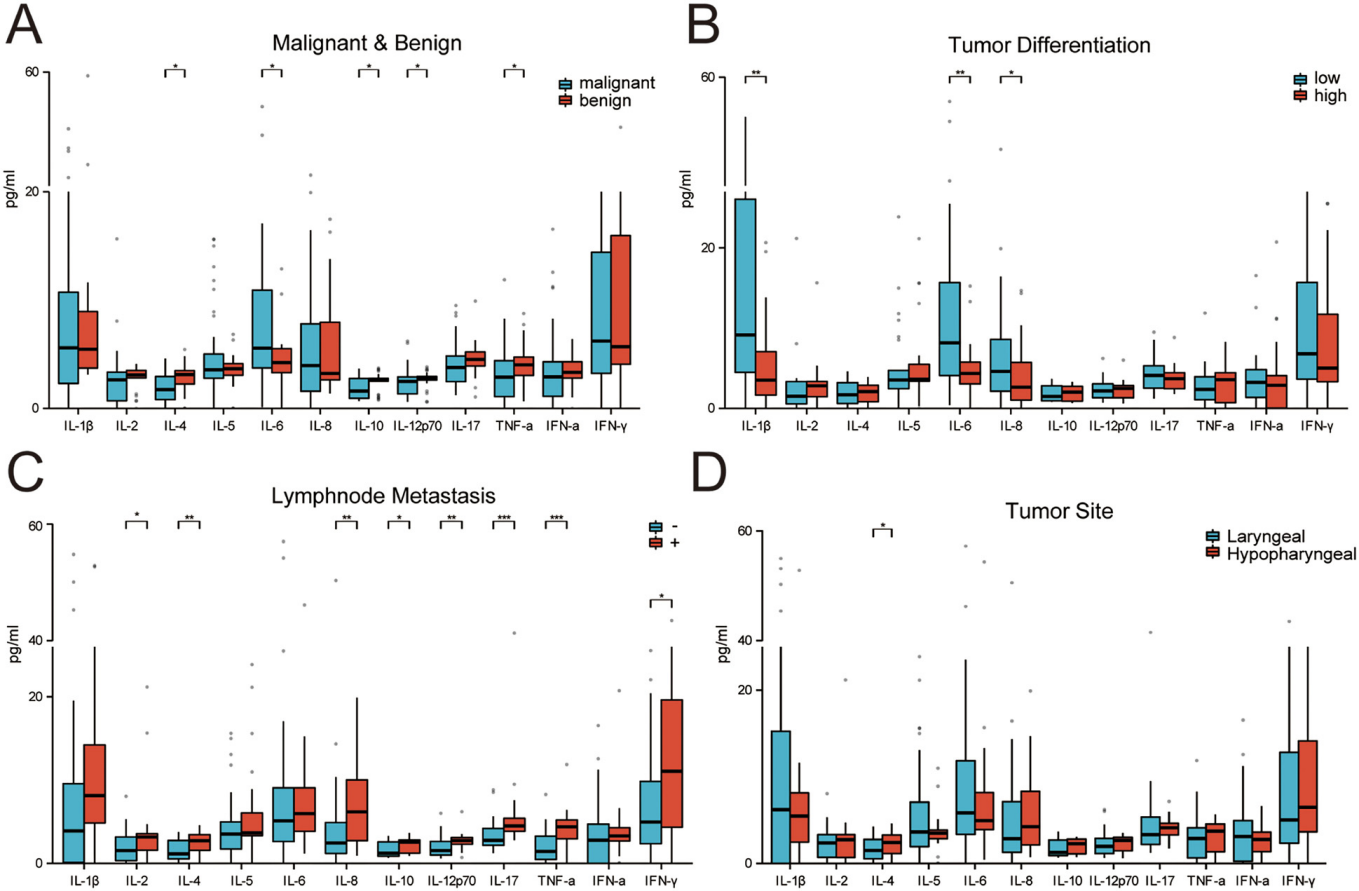
(A) Correlation between cytokines and tumor malignancy, including IL-4 (*P* ═ 0.0130), IL-6 (*P* ═ 0.0213), IL-10 (*P* ═ 0.0399), IL-12p70 (*P* ═ 0.0466), and TNF-α (*P* ═ 0.0297); (B) Correlation between cytokines and tumor differentiation, IL-1 β (*P* ═ 0.008), IL-6 (*P* ═ 0.005), and IL-8 (*P* ═ 0.05); (C) Correlation between cytokines and lymph node metastasis, IL-2 (*P* ═ 0.010), IL-4 (*P* ═ 0.028), IL-10 (*P* ═ 0.011), IL-12p70 (*P* ═ 0.034), IL-17 (*P* ═ 0.024), TNF-α (*P* ═ 0.003), IFN-γ (*P* ═ 0.007); (D) Correlation between cytokines and tumor type, IL-4 (*P* ═ 0.0048). *P < 0.05, **P < 0.01, ***P < 0.001. IL: Interleukin; TNF: Tumor necrosis factor; IFN: Interferon.

**Figure 2.**
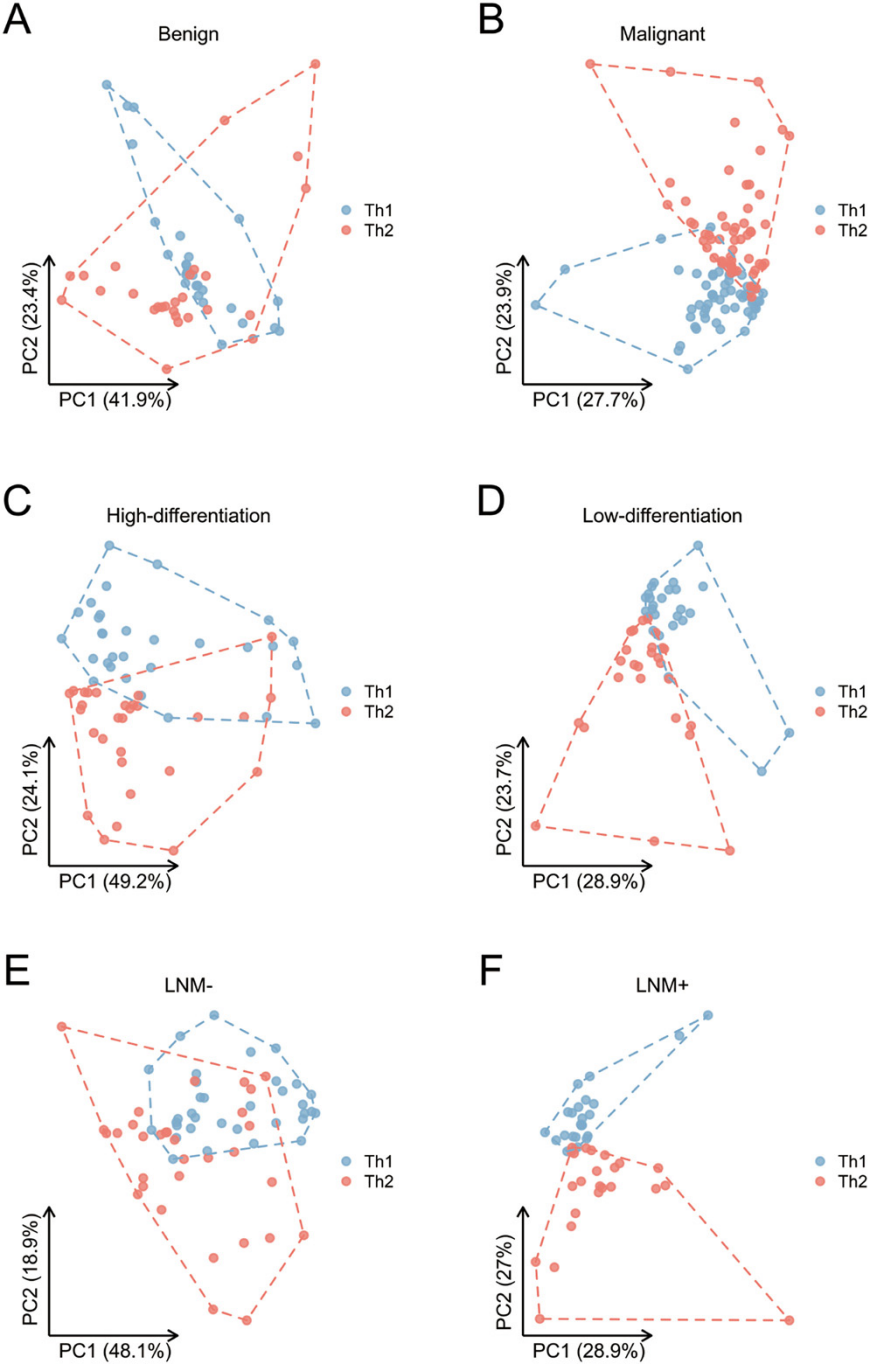
**Grouping analysis of serum 12 CKs Th1 and Th2 in patients with benign and malignant lesions showed that the malignant lesion group had a higher degree of separation between the two CKs.** Meanwhile, in the HNSCC group, the higher the degree of malignancy (low differentiation and positive lymph node metastasis), the higher the degree of separation between the two types of CKs. (A) PCA of Th1 and Th2 CKs in the benign lesion group; (B) PCA of Th1 and Th2 CKs in HNSCC group; (C) PCA of Th1 and Th2 CKs in the medium to high differentiation group; (D) PCA of Th1 and Th2 CKs in the poorly differentiated group; (E) PCA of Th1 and Th2 CKs in the negative lymph node metastasis group; (F) PCA of Th1 and Th2 CKs in lymph node metastasis positive group. CK: Cytokine; HNSCC: Head and neck squamous cell carcinoma; PCA: Principal component analysis.

In the study, the levels of serum CKs were compared between 58 pharyngeal cancer patients and 27 patients with benign vocal cord lesions. The results revealed significantly higher levels of IL-1 β, IL-6, IL-8, IFN-γ IL-2, IL-4, IL-5, IL-10, IL-17, IL-12p70, IFN-α, and TNF-α are higher in patients with tumors as compared to those with benign lesions. Notably, there was a statistically significant difference in the expression of IL-4 (*P* ═ 0.0130), IL-6 (*P* ═ 0.0213), IL-10 (*P* ═ 0.0399), IL-12p70 (*P* ═ 0.0466), and TNF-α (*P* ═ 0.0297) (Table 2). Figure 1A shows high expression of IL-6 in HNSCC, while other CKs are predominantly expressed in benign lesions. Additionally, the balance of Th1/Th2 shifted toward Th2 in tumor patients, while it leaned toward Th1 in benign patients. The results of PCA revealed a significant separation of Th1 and Th2 CKs on the PC2 axis for patients with HNSCC, but not for those with benign lesions (Figure 2A and 2B). This indicates a notable imbalance in Th1/Th2 CKs in the systemic inflammatory state of tumor patients. Altogether, patients with tumors showed high levels of immunosuppressive CKs.

**Table 2:** Correlation between serum cytokine levels (pg/mL) and different diseases

	**IL-1β**	**IL-2**	**IL-4**	**IL-5**	**IL-6**	**IL-8**	**IL-10**	**IL-12p70**	**IL-17**	**TNF-α**	**IFN-α**	**IFN-γ**
Cancer (Median)	5.585	2.63	1.735	3.545	5.56	3.945	1.59	2.47	3.77	2.875	2.9	6.205
Benign (Median)	5.46	3.08	3.12	3.65	4.21	3.22	2.67	2.82	4.51	4.01	3.32	5.69
*P*	0.6296	0.0510	0.0130	0.8933	0.0213	0.4447	0.0399	0.0466	0.0678	0.0297	0.2158	0.6515

IL: Interleukin; TNF: Tumor necrosis factor; IFN: Interferon.

**Figure 3.**
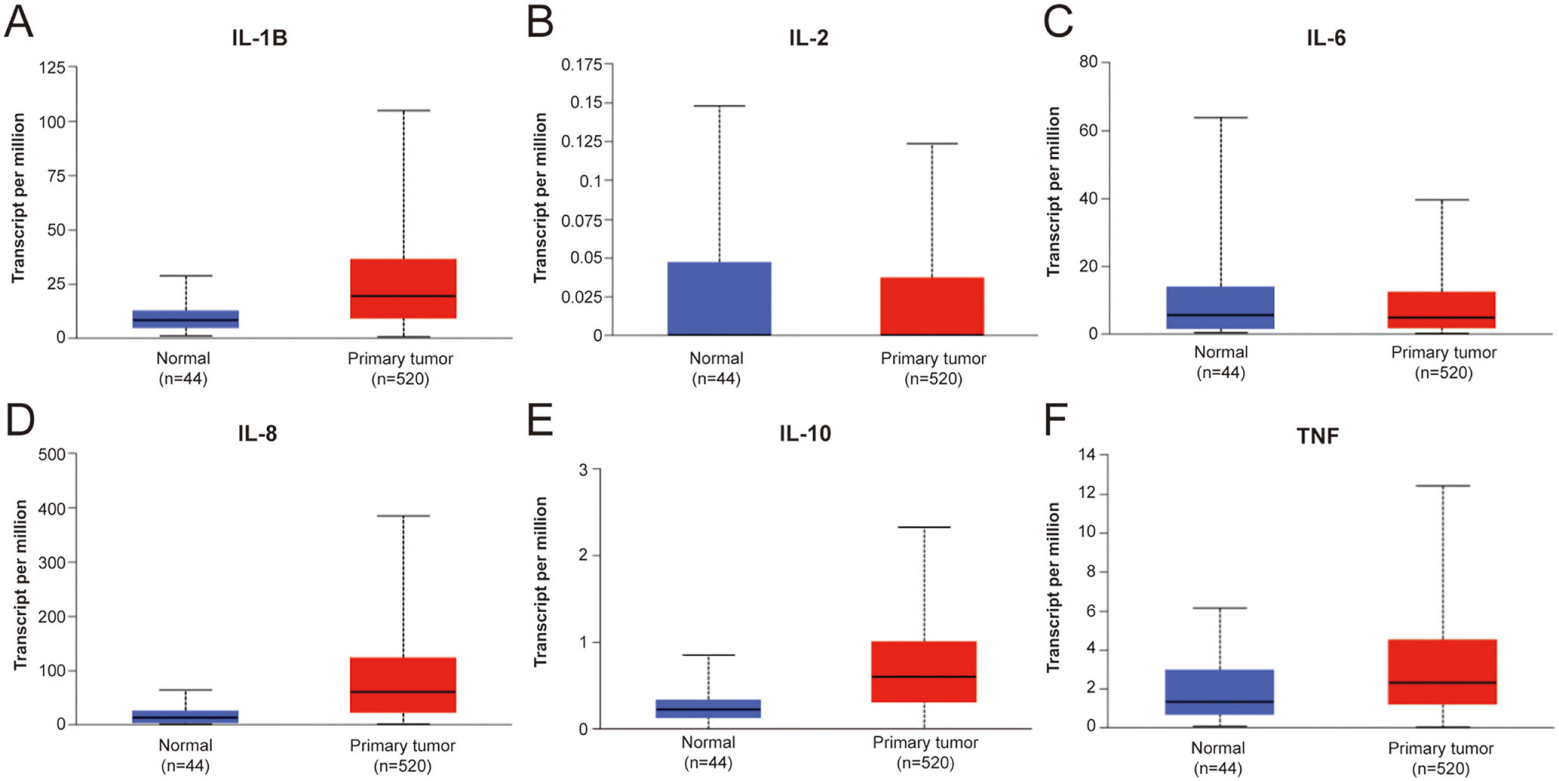
(A) IL-1β is the difference in expression between head and neck tumors and normal tissues is statistically significant, *P* < 0.01; (B) The expression difference of IL-2 in head and neck tumors and normal tissues is not statistically significant, *P* > 0.05; (C) The expression difference of IL-6 in head and neck tumors and normal tissues is not statistically significant, *P* > 0.05; (D) The expression difference of IL-8 in head and neck tumors and normal tissues is not statistically significant, *P* > 0.05; (E) The expression difference of IL-10 in head and neck tumors and normal tissues is statistically significant, *P* < 0.01; (F) The expression difference of TNF in head and neck tumors and normal tissues is statistically significant, *P* < 0.01. IL: Interleukin; TNF: Tumor necrosis factor.

When using UALCAN to examine CK expression in HNSCC from the TCGA database, it is evident that IL-1β, IL-10, and TNF are highly expressed in tumor tissues with statistical significance (*P* < 0.01). However, there were no statistically significant differences in the expression of IL-2, IL-6, and IL-8 between head and neck tumors and normal tissues (Figure 3). These results suggest that there are variations between serum CK protein levels and tissue mRNA expression levels, with consistent expression of IL-1β.

### Correlation analysis between serum CK levels and clinical characteristics of tumors

#### The correlation between serum CK levels and tumor differentiation degree

Among 58 patients diagnosed with laryngeal squamous cell carcinoma, 29 (50%) had poorly differentiated tumors. A correlation analysis was performed on the levels of 12 CKs in the patients’ serum and the degree of tumor differentiation, revealing a significant correlation between IL-1β (*P* ═ 0.008), IL-6 (*P* ═ 0.005), and IL-8 (*P* ═ 0.05) levels and the degree of tumor differentiation (Figure 1B) (Table 3). Furthermore, Th1/Th2 balance in poorly differentiated tumors favored Th2. PCA highlighted a more pronounced difference in the expression levels of the two types of CKs on the PC2 axis in the low differentiation group compared to the medium–high differentiation group (Figure 2C and 2D). The results suggest a predominant state of immunosuppression and heightened expression of associated CKs in poorly differentiated tumors.

**Table 3:** Correlation between serum cytokine levels (pg/mL) and disease characteristics

		**IL-1β**	**IL-2**	**IL-4**	**IL-5**	**IL-6**	**IL-8**	**IL-10**	**IL-17**	**IL-12p70**	**TNF-α**	**IFN-α**	**IFN-γ**
Differentiation	High differentiation	3.51	2.815	2.08	3.64	4.34	2.635	2.005	2.47	3.685	3.56	2.87	5.015
	Low differentiation	9.15	1.5	1.68	3.53	8.16	4.61	1.48	2.16	4.11	2.35	3.23	6.79
	*P*	0.0078	0.288	0.8045	0.4654	0.0045	0.0496	0.7435	0.4579	0.4579	0.5873	0.5268	0.6491
Tumor site	Laryngeal	6.2	2.365	1.49	3.63	5.835	2.85	1.255	1.965	3.32	2.87	3.095	5.015
	Hypopharyngeal	5.46	2.73	2.41	3.49	4.93	4.24	2.27	2.65	4.11	3.72	2.76	6.47
	*P*	0.8844	0.7112	0.0479	0.6581	0.7316	0.2469	0.2921	0.403	0.2996	0.3109	0.4676	0.6237
Lymph node metastasis	Yes	3.895	1.53	1.145	3.51	5.115	2.455	1.21	1.535	2.765	1.44	2.785	4.965
	No	8.13	3.17	2.715	3.66	5.97	6.19	2.555	2.735	4.5	4.39	3.295	11.06
	*P*	0.0755	0.0247	0.0049	0.1823	0.5933	0.0093	0.0167	0.0017	0.0002	0.0003	0.2727	0.0259

IL: Interleukin; TNF: Tumor necrosis factor; IFN: Interferon.

#### Analysis of differences in serum CK levels between laryngeal cancer and hypopharyngeal cancer

Among 58 patients who were diagnosed with squamous cell carcinoma of the throat, 33 had laryngeal cancer and 25 had hypopharyngeal cancer. A correlation analysis was performed on the levels of 12 CKs and the location of the tumor. The results showed (Table 3) that IL-4 (*P* ═ 0.0048) levels were significantly correlated with tumor location (Figure 1D). The expression level of IL-4 was higher in hypopharyngeal carcinoma than in laryngeal carcinoma, and this immunosuppressive state may be related to a poorer prognosis in nasopharyngeal carcinoma.

#### Correlation between serum CK levels and tumor lymph node metastasis

Among 58 patients, 25 (43.10%) had lymph node metastasis. A correlation analysis was conducted between 12 CK levels and lymph node metastasis. The results indicated a significant correlation between IL-2 (*P* ═ 0.025), IL-4 (*P* ═ 0.005), IL-8 (*P* ═ 0.009), IL-10 (*P* ═ 0.017), IL-12p70 (*P* ═ 0.002), IL-17 (*P* < 0.001), TNF-α (*P* < 0.001), and IFN-γ (*P* ═ 0.026) and lymph node metastasis (Figure 1C). Multiple logistic regression was used to compare these seven CKs to other factors that may affect lymph node metastasis, including age, gender, smoking and drinking history, location, T stage, and differentiation. The results showed that there is a statistically significant correlation between the expression level of IL-2 (*P* ═ 0.010), IL-4 (*P* ═ 0.028), IL-10 (*P* ═ 0.011), IL-12p70 (*P* ═ 0.034), IL-17 (*P* ═ 0.024), TNF-α (*P* ═ 0.003), and IFN-γ (*P* ═ 0.007) and lymph node metastasis (Table 3). The PCA results exhibited significant differences in the expression levels of Th1 and Th2 CKs on the PC2 axis in Figure 2E and 2F between the lymph node metastasis negative and positive groups. The overall expression of CKs was markedly higher in patients with lymph node metastasis, indicating a link to tumor progression and mobilization of cellular and humoral immunity in the body.

The study found no significant correlation between the levels of 12 CKs in the patient’s serum and their tumor stage.

### Serum CKs and neoadjuvant therapy

**Figure 4.**
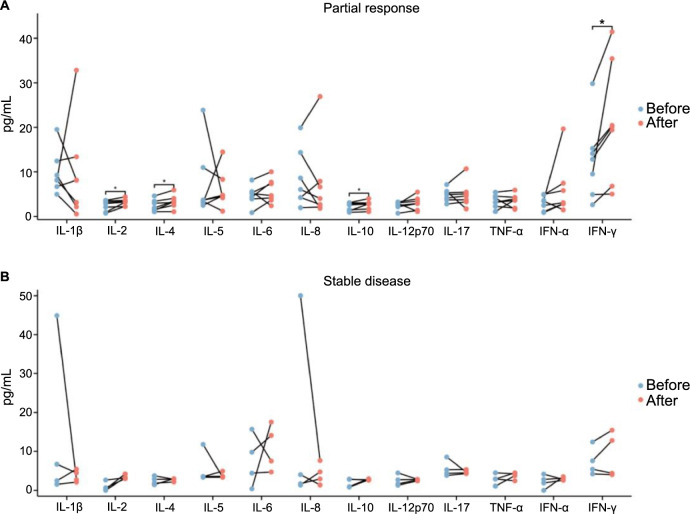
(A) Paired map of serum cytokine levels before and after immunotherapy in PR group patients; (B) Paired map of serum cytokine levels before and after immunotherapy in SD group patients. IL-2 (*P* ═ 0.016), IFN-γ (*P* ═ 0.016), IL-4 (*P* ═ 0.016), IL-10 (*P* ═ 0.031). *P < 0.05. SD: Stable disease; IL: Interleukin; IFN: Interferon; PR: Partial response; TNF: Tumor necrosis factor.

Among 58 patients, 11 of them received neoadjuvant therapy comprising pembrolizumab and chemotherapy. Among them, five patients were treated for two cycles and six patients for one cycle. Seven patients had PR and four patients had SD. The serum levels of 12 CKs were measured before and after immunotherapy. The results showed that the expression levels of IL-2 (*P* ═ 0.016) and IFN-γ (*P* ═ 0.016), IL-4 (*P* ═ 0.016), and IL-10 (*P* ═ 0.031) in the PR group were significantly increased before treatment (Table 4 and Figure 4), while there was no significant difference in the 12 CKs before and after immunotherapy in the SD group patients.

**Table 4:** Correlation analysis between the efficacy of neoadjuvant therapy and changes in serum cytokine levels (pg/mL)

**Grouping**		**IL-1β**	**IL-2**	**IL-4**	**IL-5**	**IL-6**	**IL-8**	**IL-10**	**IL-17**	**IL-12p70**	**TNF-α**	**IFN-α**	**IFN-γ**
PR	Before (Median)	8.13	2.85	1.87	3.64	5.1	6.06	2.56	2.74	4.87	3.15	3.41	12.8
	After (Median)	8.13	3.2	3.31	4.64	4.69	3.99	2.79	2.87	4.59	3.74	3.06	20.2
	*P*	0.5781	0.0156	0.0156	0.8125	0.375	0.6875	0.0313	0.2188	0.271	0.4688	0.1563	0.0156
		**IL-1β**	**IL-2**	**IL-4**	**IL-5**	**IL-6**	**IL-8**	**IL-10**	**IL-17**	**IL-12p70**	**TNF-α**	**IFN-α**	**IFN-γ**
SD	Before (Median)	4.57	0.49	2.32	3.52	7.1	2.86	1.88	2.17	4.83	2.9	2.34	6.49
	After (Median)	3.6	3.24	2.84	3.48	10.8	3.83	2.69	2.76	4.6	4	2.95	8.6
	*P*	0.875	0.125	0.625	1	0.625	0.875	0.625	0.875	0.875	0.625	0.625	0.625

IL: Interleukin; TNF: Tumor necrosis factor; IFN: Interferon; SD: Stable disease; PR: Partial response.

### The correlation between serum CK levels and tumor markers

A correlation analysis was conducted between the levels of 12 CKs and tumor markers in 58 patients. The results showed that IL-2 (*P* ═ 0.002), IL-6 (*P* ═ 0.036), IL-10 (*P* ═ 0.022), IL-12p70 (*P* ═ 0.042), and IFN-α (*P* ═ 0.001) were significantly negatively correlated with the positive expression of p16 in tumor tissue; IL-2 (*P* ═ 0.039) was significantly negatively correlated with positive expression of p63 in tumor tissue; IL-8 (*P* ═ 0.028) was significantly positively correlated with the expression of Ki-67 in tumor tissue; the level of IFN-α (*P* ═ 0.013) is significantly positively correlated with the CPS index of PD-L1in peripheral blood. This association between PD-L1 expression and its level suggests its potential role in anti-tumor immunity (Table 5).

**Table 5:** Correlation analysis between serum cytokines and markers

		**IL-1β**	**IL-2**	**IL-4**	**IL-5**	**IL-6**	**IL-8**	**IL-10**	**IL-12p70**	**IL-17**	**TNF-α**	**IFN-α**	**IFN-γ**
P16	Z	−1.738	−3.064	−1.746	−0.931	−2.092	−1.371	−2.286	−2.036	−1.316	−2.037	−3.267	−1.275
	P	0.082	0.002	0.081	0.352	0.036	0.17	0.022	0.042	0.188	0.042	0.001	0.202
P63	Z	−0.999	−2.062	−1.113	−0.814	−0.063	−0.908	−1.224	−0.513	−0.979	−1.547	−1.473	−0.513
	P	0.318	0.039	0.266	0.416	0.95	0.364	0.221	0.608	0.328	0.122	0.141	0.608
Ki-67	Z	0.048	0.013	0.141	0.093	−0.037	0.291	0.098	0.068	0.098	−0.02	0.161	0.203
	P	0.721	0.925	0.295	0.495	0.783	0.028	0.468	0.616	0.468	0.883	0.23	0.13
PD-L1(CPS)	Z	0.157	0.115	0.106	−0.094	0.279	0.108	0.085	0.118	−0.081	0.152	0.433	0.333
	P	0.389	0.533	0.564	0.616	0.122	0.557	0.645	0.522	0.658	0.406	0.013	0.062

IL: Interleukin; TNF: Tumor necrosis factor; IFN: Interferon; PD-L1: Programmed death ligand 1; CPS: Combined positive score.

## Discussion

According to previous research, CKs can be categorized as proinflammatory or anti-inflammatory, depending on their impact on inflammation [2]. Pro-inflammatory CKs include IL-1β, IL-6, and TNF-α, while anti-inflammatory ones include IL-4, IL-10, and IL-17, among others. The main source of CKs is T lymphocytes, and specifically, CD4+T lymphocytes, also known as helper T cells (Th cells), which are the primary CK producers. Mosmann and Sad [3] described CD4+Th cell subpopulations and named them TH1, TH2, TH9, TH17, TH22, or follicular TH cells based on the different CKs they produce. These T cell subsets can promote different types of inflammatory responses based on their respective CK profiles, responses to chemokines, and interactions with other cells. Th1 CKs mainly include IFN-γ, TNF-α, TNF-β, and CKs, such as IL-2 and IL-12; these CKs can promote T cell-mediated immune response, which is cellular immunity, releasing CKs that cause inflammation and mediating cellular immunity. Th2 CKs mainly include CKs such as IL-4, IL-6, and IL-10; the main function of these CKs is to mediate the humoral immune response and inhibit the Th1 response. When the body is in a normal state, the number of Th1 and Th2 cells is in a dynamic equilibrium state. Th1 and Th2 secrete CKs, cross-regulate and inhibit each other, maintain normal cellular and humoral immune functions, and maintain a balance in the human immune system. When the body experiences functional abnormalities, Th1 and Th2 often exhibit a balance bias toward one side, known as “Th1/Th2 drift.” The balance state is disrupted, causing the dynamic balance of the human CK network to be disrupted. In the body of tumor patients, there is a balance drift of Th1/Th2. When Th2 CKs dominate, tumor immune escape occurs.

Research has shown that changes in CK expression play an important role in the malignant transformation of many cancers, including HNSCC [2]. Due to the excessive production of some CKs by tumor cells, they can serve as important diagnostic markers in the serum of HNSCC patients; thus, their research value is gradually highlighted. Evidence shows that HNSCC is linked to a decrease in Th1 CKs and an increase in Th2 CKs, which allows tumors to evade anti-tumor immune responses and stimulate tumor growth [4]. This shift toward a Th2 CK response is a common phenomenon in other solid tumors, such as colorectal cancer, renal cell carcinoma, prostate cancer, and melanoma. Our study found significantly higher levels of Th1 CKs IL-12p70 and TNF-α in benign lesions compared to patients with pharyngeal squamous cell carcinoma. On the other hand, the expression levels of Th2 CKs IL-6 and IL-8 were higher in pharyngeal squamous cell carcinoma than in benign lesions, consistent with previous literature [5].

In this study, we also found that IL-4 and IL-10, as members of the TH2 class of CKs, were more expressed in the benign lesion group. At the same time, in immunotherapy patients who responded well, IL-4 and IL-10 significantly increased after treatment. This is contrary to the previously agreed conclusion. However, there are currently some cancer-related studies, including HNSCC, that have demonstrated the biological functions of these two CKs that differ from other Th2 CKs. Hoffmann et al. [6] found that the serum levels of IL-4 and IL-10 did not significantly increase in 20 patients with HNSCC, compared to a control group of 20 patients. Similarly, in a study involving 93 patients with HNSCC and 53 healthy controls, no significant increase of IL-10 was observed in the malignant patient group [7]. Some literature suggests that although IL-4 has been previously linked to promoting tumors, it may also have anti-tumor effects, depending on its level and interactions with other immune regulatory factors [8]. A study on advanced HNSCC showed that the expression level of IL-4 was closely correlated with IL-2 and IFN-γ [9]. Additionally, some researchers speculate that the specific dynamics (half-life), metabolism, and orbital protein regulatory parameters of each individual HNSCC tumor could explain these contradictory findings [10].

The high secretion level of CKs also participates in the indirect regulation of immune response and tumor cell differentiation process [11]. Among them, dysregulation of IL-6 signaling usually plays a core role in the differentiation of HNSCC tumor cells [12]. Meanwhile, IL-1β also plays an important role in the differentiation of laryngeal squamous cell carcinoma. We know that one of the biological effects of IL-1 is to trigger an increase in the expression of IL-6, etc. Investigations into the development of HNSCC have revealed a positive correlation between serum levels of IL-1β and tumor staging, as well as its potential to promote cell proliferation, colony formation, and tumorigenicity. It can promote the stem cell characteristics of HNSCC cells by activating the Smad/ID1 signaling pathway [13]. In this study, it was also demonstrated that IL-1β-IL-6, and IL-8 are abnormally overexpressed in poorly differentiated laryngeal squamous cell carcinoma, indicating a significant difference compared to the control group.

IL-8, a proinflammatory CK belonging to the CXC chemokine family, has been linked to cancer invasion, angiogenesis, and metastasis [14, 15]. In certain cancers, such as breast cancer, colon cancer, cervical cancer, pancreatic cancer, and leukemia, it has been established that IL-8 can be secreted by cancer cells themselves or through paracrine signaling. Over the past decade, four studies have investigated the role of IL-8 in LSCC, enrolling a total of 220 patients with LSCC [16–19]. These studies have found that elevated levels of IL-8 CKs in cancer patients are primarily associated with tumor size and lymph node metastasis [17]. Recent studies have shown that lymph node metastasis can accelerate the hematogenous metastasis of tumors by affecting systemic immune surveillance [20]. In human lymph node metastasis, high levels of MHC-II expression have been observed and are linked to associated signals. Some experts postulate that the entry of tumor cells into lymph nodes induces IFN-gamma, which triggers signal transduction and upregulates MHC-II, leading to local immune suppression caused by Treg infiltration and enhancing LN seeding [21]. Researchers selected malignant cells from squamous cell carcinoma of the head and neck, and compared the transcriptome of lymph node metastasis and non-lymph node metastasis. They found significant changes in IFN-related genes [20]. In this study, IFN-γ was assessed through single-factor and multiple-factor analysis and was found to be notably higher in patients with HNSCC and lymph node metastasis, further supporting the notion of local immune suppression in lymph node metastasis and immune editing of lymph nodes following tumor cell invasion.

Lathers and Young [9] were the first to report differences in CK changes in HNSCC, finding a correlation with primary tumor subpopulations. IL-10 expression is highest in nasopharyngeal carcinoma, followed by laryngeal carcinoma. However, Kaskas et al. [22] believed that the expression of specific serum CKs in HNSCC patients is consistent regardless of the tumor site. This study demonstrates that compared to laryngeal cancer, IL-4, as a classic Th2 class CK, is highly expressed and has significant differences in hypopharyngeal cancer. The high expression of its immunosuppressive CKs may be one of the reasons for the poorer prognosis of hypopharyngeal cancer compared to laryngeal cancer.

Serum CKs as molecular markers have been highly valued by researchers in evaluating the efficacy of tumor immunotherapy [1]. A research team retrospectively analyzed 1344 phase III clinical trials of advanced cancer treated with anti-PD-1 antibodies and anti-CTLA-4 antibodies. The results showed that high levels of serum IL-8 expression were associated with poor cancer prognosis and reduced clinical efficacy [23]. Laino et al. [24] analyzed the serum of patients from three large phase II/III randomized ICI trials and found that higher baseline IL-6 levels were associated with shorter survival. Some studies have also demonstrated that patients who experience tumor remission after treatment with anti-PD-1 monoclonal antibodies show an increase in IFN expression, suggesting a correlation with improved immunotherapy effectiveness [8].

Overall, this study focused on detecting the expression of PD-L1 in tumor tissue from patients with pharyngeal squamous cell carcinoma. The CPS was used, and its correlation with CKs in the patient’s serum was analyzed. Results demonstrated a significant relationship between PD-L1 expression in the serum and CPS, which also showed a connection to the effectiveness of neoadjuvant therapy. Prior studies have revealed that IFN-α plays a role in PD-L1 regulation through the activation of the PI3K-AKT-mTOR signaling pathway and is involved in IFN-dependent mRNA transcription. However, there is a limited amount of literature on this topic concerning laryngeal squamous cell carcinoma. This article marks the first report on the significant correlation between CPS and PD-L1 expression. The JAKs/STATs/IRF1 axis serves as the main mode of regulation for PD-L1 expression, with type II IFN being the primary inducer. This pathway has also been implicated in various forms of cancer, including melanoma, non-small cell lung cancer, liver cancer, HNSCC, and gastric cancer [25, 26]. Serum IFN levels may serve as a potential biomarker for identifying the progression of anti-PD-1 monoclonal antibody therapy in HNSCC, although more immunotherapy samples are required to confirm this finding.

## Conclusion

To conclude, this study suggests that patients with pharyngeal tumors exhibit a skewed Th1/Th2 balance toward Th2, with a greater separation between Th1 and Th2 CKs in those with HNSCC, indicative of increased malignancy. Future studies should aim to utilize this CK ratio as a reliable measure for clinical and pathological assessment of cancer patients. Moreover, IFN shows promise as a potential circulating molecular marker for evaluating the effectiveness of immunotherapy in HNSCC.

## Data Availability

The datasets used and analyzed during the current study are available from the corresponding author on reasonable request.
